# Outcome predictors in anterior and posterior ischemic strokes: a study based on the Iranian SITS registry

**DOI:** 10.1038/s41598-023-28465-8

**Published:** 2023-01-21

**Authors:** Nazanin Jalali, Elyar Sadeghi Hokmabadi, Abdoreza Ghoreishi, Payam Sariaslan, Shahram Rafie, Afshin Borhani-Haghighi, Amir Moghadam Ahmadi, Hossein Azin, Alireza Vakilian, Parvin Khalili, Mehdi Farhoudi

**Affiliations:** 1 Department of Neurology, School of Medicine, Non-Communicable Diseases Research Center, Rafsanjan University of Medical Sciences, Rafsanjan, Iran; 2 Neurosciences Research Center, Neurology Department, Tabriz University of Medical Sciences, Tabriz, Iran; 3 Stroke Research Group, Vali-E-Asr Hospital and Department of Neurology and Stroke Unit, School of Medicine, Zanjan University of Medical Sciences, Zanjan, Iran; 4 Clinical Research Development Center, Imam Reza Hospital, Kermanshah University of Medical Sciences, Kermanshah, Iran; 5 Department of Neurology, School of Medicine, Ahvaz Jundishapur University of Medical Sciences, Ahvaz, Iran; 6 Clinical Neurology Research Center, Shiraz University of Medical Sciences, Shiraz, Iran; 7 Department of Epidemiology, School of Public Health, Social Determinants of Health Research Center, Rafsanjan University of Medical Sciences, Rafsanjan, Iran

**Keywords:** Diseases, Neurology

## Abstract

Ischemic stroke is the major form of stroke with two separate vascular territories. Many risk factors are related to stroke outcomes in both territories. The present descriptive research was carried out on the basis of data obtained from the Safe Implementation of Treatments in Stroke (SITS) registry on Iranian intravenous thrombolysis ischemic stroke cases. Vascular territory involved in each case and three-month excellent outcome, functional independence, mortality rate, and brain hemorrhage occurrence were determined. Univariable and multivariable logistics regression analyses were utilized in order to investigate association of ischemic stroke outcomes with the vascular territory involved and other related factors. Among 1566 patients 95.4% was anterior circulation stroke patients and 4.6% was posterior circulation stroke cases. There is no significant association between vascular territory with mortality (OR of PCS vs ACS: 0.74, 95% CI 0.37–1.46), excellent functional outcome (OR 0.72, 95% CI 0.44–1.19), functional outcome (OR 0.86, 95% CI 0.52–1.42) and local hemorrhage (OR 0.98, 95% CI 0.30–3.21). Among major risk factors, age, diabetes, NIHSS score and admission duration, increased significantly odds of three-month mortality, excellent outcome, and functional independence in the multivariate analysis. The highest of odds was in NIHSS score with a dose–response association. The vascular territory was not an outcome predictor in ischemic strokes. The most important predictor was baseline NIHSS.

## Introduction

After ischemic heart disease, stroke is the second-leading cause of death globally. It was also the third-leading cause of death and disability in 2019, after newborn illnesses and ischemic heart disease.

In 2019, ischemic stroke accounted for 62.4% of all new strokes. From 1990 to 2019, its burden (in terms of the total case number) rose dramatically^1^.

Ischemic stroke is divided into two types based on the vascular areas where ischemic lesions occur: anterior circulation stroke (ACS) and posterior circulation stroke (PCS). The former includes part of the carotid artery system, the internal carotid artery. The latter consists of the basilar and vertebral arteries and their branches^2^.

The stroke outcome is related to many risk factors. Vascular territory as a factor in the IVT outcome has had contradictory results in various studies. In some studies, the results have shown a reduction in the risk of bleeding, a higher rate of functional improvement, and excellent recovery in PCSs; however, the mortality rate has not been different compared to ACSs^3^.

According to another study, there is no difference in safety and effect between ACS and PCS in delayed IVT therapy; however, a trend toward less bleeding, not statistically significant, is reported in the posterior strokes^4,5^.

Lu et al., in their study of wake-up stroke, found that the circulation as anterior versus posterior, unlike the National Institutes of Health Stroke Scale (NIHSS) and Atrial Fibrillation (AF), did not affect the outcome^6^. Bensten et al. related uncontrolled blood pressure to poor outcomes in patients who received IVT therapy after an ischemic stroke^7^.

Kim et al. described that minor PCS was substantially related to more disability at 3 months than minor ACS^8^.

According to Handelsman et al., ACS cases with aging had a worse prognosis than those of PCS. They showed NIHSS as an essential predicting factor for the functional state in both territories, with no difference in their outcomes^9^.

Many studies suggest similar outcomes for both stroke regions^10–16^.

The present study aims to investigate prognostic factors in Iranian IVT therapy cases registered in the SITS registry, especially in stroke territory, as a critical factor in the characteristics and presentation of stroke.

## Methods

### Data collection and patients

The present research was retrospective registry-based, in which the possible influencing factors, especially the circulation type involved, were analyzed for the outcomes of ischemic stroke patients who received IVT. Globally, the Safe Implementation of Thrombolysis in Stroke (SITS) was the largest stroke thrombolysis registry, showing the safety of IVT (2008)^17^. As a part of this registry, the current study compared the outcome of IV thrombolysis in ACSs and PCSs in Iranian cases registered in SITS since vascular territory is a challenging factor in the outcome of IVT therapy cases.

This study used data from the SITS, International Stroke Thrombolysis Register—intravenous standard therapy section (ISTR-IVST), of five Iranian hospital cases between june 2011 and October 2021. Zanjan, Tabriz, Kermanshah, Ahvaz, and Rafsanjan centers were involved in this study. SITS-ISTR data collection and administration procedure have already been explained in detail^3,18–20^. All methods were carried out following the relevant guidelines and regulations of SITS and ISTR-IVST^3,18–20^.

In this study, all cases registered in the Iranian ISTR-IVST of SITS registry as ischemic stroke patients who have completed data about the type of territory involved and 3 months functional state were included. All data collection was performed by experienced neurologists in these hospitals. Clinical data were collected at admission, discharge, and 3-month follow-up time in person or by telephone.

Data included baseline demographics, stroke risk factors, pre-stroke functional status (as obtained by the modified Rankin Scale (mRS) [score 0–6], stroke severity (as determined by NIHSS score 0–42), admission time, drug treatment history, needle time, and complications. For all IVT cases, 24-h imaging scan data were provided. The ICD-10 code offered data on etiological subtypes of a stroke at discharge time as other causes for admission, such as stroke mimics, were not included. At three months, the last follow-up with mRS was obtained. The outcome of patients with IVT therapy was compared in ACS and PCS. Other possible risk factors were also checked in their outcome.

### Outcomes

- Functional independence was one outcome at the 3-month follow-up, as judged by an mRS score ≤ 2.
- Excellent outcome was determined as an mRS score ≤ 1 at 3 months.
- Mortality rate within 3 months of follow-up was considered as another outcome.
- Hemorrhage, bleeding at the stroke site or other part of the brain, was another outcome or complication in this study. The hemorrhagic transformation is divided into hemorrhagic infarction (HI) and parenchymal hematoma (PH) by European Cooperative Acute Stroke Study II (ECASSII) standard. Symptomatic intracranial hemorrhage (SICH) is considered a worse condition as NIHSS score > 4 or ≥ 2 points of a subcategory NIHSS due to the hemorrhagic effect^21^. The present study combined bleeding of HI and PH as local hemorrhage due to a small number of cases.

To learn more about the vascular territory impact on the IVT patient outcome, the outcome endpoints of those who received IVT in ACS were compared to the outcome endpoints of PCS patients. Other important factors, including sex, age, diabetes, hypertension, hyperlipidemia, cigar-smoking, the severity of strokes as NIHSS score, AF, onset-to-needle time, duration of admission, and type of stroke, were investigated in relation to outcome endpoints alongside the vascular territory.

### Covariate measurements

Some of the covariate measurements need descriptions as below:

- Vascular territory is a factor that points to two kinds of stroke (ACS and PCS) based on the anterior and posterior circulation of the brain.
- Hypertension is defined by diastolic blood pressure ≥ 90, systolic blood pressure ≥ 140, or a history of antihypertensive drug use.
- Diabetes mellitus is considered as a history of antidiabetic drug use, fasting blood glucose level ≥ 126 mg/dl, or blood glucose level ≥ 140 mg/dl 2 h postprandial.
- NIHSS is considered a scale for calculating the degree of some important abilities that show stroke severity.
- Rankin modified scale (mRS), with six grades, shows the functional ability of patients.
- Type of stroke is determined by a CT scan and MRI of the brain, as well as a vascular and cardiac evaluation, to determine the form of stroke event (as the large vessel, other large vessel, embolic, lacunar, and other types).

### Statistical analyses

Quantitative variables were expressed as the mean ± standard deviation or median Interquartile range [IQR], and categorical variables were expressed as the frequency and percentage. Further, patient baseline characteristics were compared in the circulation type groups (ACS and PCS) and outcomes of ischemic stroke patients who received IVT, utilizing the chi-square test for categorical variables, the Mann–Whitney U test for non-normally distributed quantitative variables, and independent t-test for normally distributed quantitative variables.

Furthermore, univariable and multivariable logistics regression analyses were used to define the odds ratios (ORs) and the corresponding 95% confidence intervals (CI) for the ischemic stroke outcome's association with the circulation type involved and other related factors. Thus, at the bivariate level, separate models were run first to acquire variables related to ischemic stroke outcomes. Variables with a p-value < 0.25 were then included in the multivariable logistic regression models to obtain the 95% confidence interval (CI) and odds ratio (OR). Hosmer–Lemeshow test was used to assess the goodness-of-fit of the adjusted model.

All analyses were carried out utilizing State V.12. All p-values were two-sided; further, p-values < 0.05 and 95% confidence intervals were regarded as statistically significant.

### Ethical consideration

All stroke patients or their legal guardians provided informed consent in admission time. Patient data are used in various researches by code of the patient without private data after that study-protocol approval by the official review committee. The ethical committee of Rafsanjan University of Medical Sciences, Rafsanjan, Iran, approved this study (NO: IIR.RUMS.REC.1399.199). Data were extracted from SITS registry.

## Results

**Figure 1:**
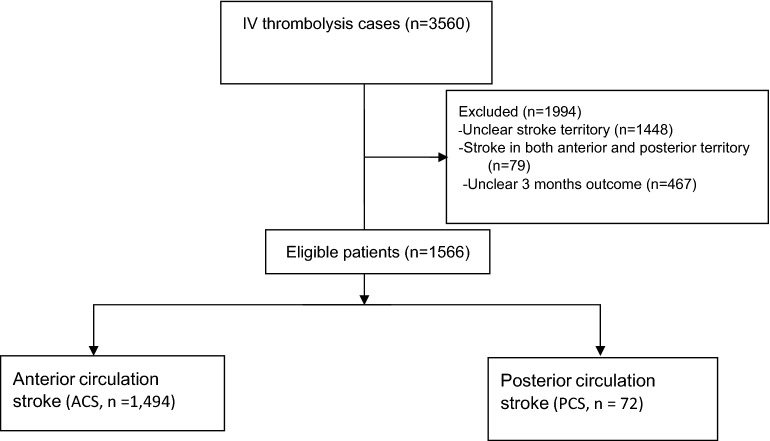
Flow chart of study.

Out of 3560 patients enrolled in Iranian hospitals in SITS registry between June 2011 and October 2021, the data of 1566 were analyzed in the present study: 1494 (95.40%) in the ACS group and 72 (4.60%) in the PCS group (Fig. 1). Table 1 presents baseline characteristics of the groups.

**Table 1:** Baseline characteristics of study population according to circulation territory.

	Total (n = 1566)	ACS (n = 1494)	PCS (n = 72)	P-value
Age—year. Mean ± SD	67.49 ± 13.89	67.51 ± 13.84	67.07 ± 14.90	0.791
Gender-no. (%)				
Male	840 (53.64)	802 (53.68)	38 (57.78)	0.881
Female	726 (46.36)	692 (46.32)	34 (47.22)	
Hypertension-no. (%)				
Yes	1361 (87.19)	1301 (87.37)	60 (83.33)	0.316
No	200 (12.81)	188 (12.63)	12 (16.67)	
Diabetes mellitus-no. (%)				
Yes	761 (51.77)	723 (51.46)	38 (58.46)	0.269
No	709 (48.23)	682 (48.54)	27 (41.54)	
Hyperlipidemia-no. (%)				
Yes	178 (11.46)	170 (11.47)	8 (11.27)	0.958
No	1375 (88.54)	1312 (88.53)	63 (88.73)	
Atrial fibrillation-no. (%)				
Yes	218 (13.97)	208 (13.96)	10 (14.8)	0.976
No	1343 (86.03)	1282 (86.04)	61 (85.92)	
Cigarette smoking-no. (%)				
Yes	158 (10.15)	155 (10.44)	3 (4.17)	0.085
No	1399 (89.85)	1330 (89.56)	69 (95.83)	
NIHSS baseline score. Mean ± SD	11.42 ± 6.91	11.56 ± 6.93	8.04 ± 5.66	< 0.001
Onset to thrombolysis-min. Mean ± SD	142.95 ± 60.26	142.92 ± 60.34	143.48 ± 59.03	0.941
Duration of admission. Median (IQR)	5 (3–9)	5 (3–10)	5 (3–8)	0.801
Door to needle-min. Mean ± SD	42.74 ± 35.60	42.30 ± 35.43	51.68 ± 37.98	0.030
Type of Ischemic stroke-no. (%)				
Large vessel	220 (14.11)	205 (13.78)	15 (21.13)	0.159
Other large	790 (50.67)	760 (51.08)	30 (42.25)	
Embolic	107 (6.86)	104 (6.99)	3 (4.23)	
Small	194 (12.44)	181 (12.16)	13 (18.31)	
Other	248 (15.91)	238 (15.99)	10 (14.08)	

The frequency difference between total number and some of the covariates was related to missing data.

In patients with ACS, the means of arterial pressure (MAP) (109.05 ± 15.64 vs. 103.31 ± 14.89), and baseline diastolic blood pressure (87.55 ± 12.17 vs. 82.07 ± 11.45) were significantly higher than PCS cases (not mentioned in the table).

NIHSS baseline was lower in PCS cases significantly. Door-to-needle time was longer in PCS cases significantly (Table 1).

Among all participants, 17.75% had 3-month mortality, 40.61% had a 3-month excellent outcome, 35.25% had a 3-month functional independence, and 4.55% had local hemorrhage (Table 2).

**Table 2:** Association of patient characteristics with outcome of IVT ischemic stroke.

	Mortality (n = 278)			Excellent functional (n = 636)			Functional outcome (n = 552)			Local hemorrhage (n = 67)		
	Yes (%)	No (%)	p	Yes	No	p	Yes	No	p	Yes	No	p
Age-year. no (%)												
< 70	737 (57.22)	80 (28.78)	< 0.001	586 (63.01)	231 (36.32)	< 0.001	633 (62.43)	184 (33.33)	< 0.001	28 (41.79)	745 (53.06)	0.071
≥ 70	551 (73.56)	198 (71.22)		344 (36.99)	405 (63.68)		381 (37.57)	368 (66.67)		39 (58.21)	659 (46.94)	
Gender.no (%)												
Male	144 (51.80)	696 (54.04)	0.497	554 (54.64)	286 (51.81)	0.284	508 (54.62)	332 (52.20)	0.345	40 (59.70)	750 (53.42)	0.314
Female	134 (48.20)	592 (55.96)		460 (45.36)	266 (48.19)		422 (45.38)	304 (47.80)		27 (40.30)	654 (46.58)	
Vascular territory.no (%)												
ACS	268 (96.40)	1226 (95.19)	0.380	882 (94.84)	612 (96.23)	0.198	965 (95.17)	529 (95.83)	0.548	64 (95.52)	1340 (95.44))	0.975
PCS	10 (3.60)	62 (4.81)		48 (5.16)	24 (3.77)		49 (4.83)	23 (4.17)		3 (4.48)	64 (4.56)	
Hypertension.no (%)												
Yes	254 (92.36)	1107 (86.08)	0.005	794 (85.47)	567 (89.72)	0.014	863 (85.19)	498 (90.88)	0.001	55 (83.33)	1230 (87.79)	0.283
No	21 (7.64)	179 (13.92)		135 (14.53)	65 (10.28)		150 (14.81)	50 (9.12)		11 (16.67)	171 (12.21)	
Diabetes mellitus.no (%)												
Yes	160 (63.75)	601 (49.30)	< 0.001	408 (46.15)	353 (60.24)	< 0.001	452 (47.08)	309 (60.59)	< 0.001	35 (56.45)	680 (51.44)	0.440
No	91 (36.25)	618 (50.70)		476 (53.85)	233 (39.76)		508 (52.92)	201 (39.41)		27 (43.55)	642 (48.56)	
Atrial fibrillation												
Yes	56 (20.51)	162 (12.58)	0.001	106 (11.40)	112 (17.75)	< 0.001	118 (11.64)	100 (18.28)	< 0.001	21 (32.81)	182 (12.98)	< 0.001
No	217 (79.49)	1126 (87.42)		824 (88.60)	519 (82.25)		896 (88.36)	447 (81.72)		43 (67.19)	1220 (87.02)	
Cigarette smoking												
Yes	21 (7.66)	137 (10.68)	0.134	99 (10.68)	59 (9.37)	0.399	109 (10.79)	49 (8.96)	0.253	6 (9.38)	138 (9.87)	0.896
No	253 (92.34)	1146 (89.32)		828 (89.32)	571 (90.63)		901 (89.21)	498 (91.04)		58 (90.63)	1260 (90.13)	
NIHSS baseline score												
Low (< 6)	10 (4.10)	309 (28.85)	< 0.001	269 (35.30)	50 (6.09)	< 0.001	283 (33.57)	36 (7.63)	< 0.001	3 (6.38)	302 (25.21)	< 0.001
Low-middle (6–14)	68 (27.87)	501 (46.78)		369 (48.43)	200 (36.17)		414 (49.11)	155 (32.84)		10 (21.28)	534 (44.57)	
Middle-high (15–24)	145 (59.43)	233 (21.76)		113 (14.83)	265 (47.92)		133 (15.78)	245 (51.91)		29 (61.70)	322 (26.88)	
High (≥ 25)	21 (8.61)	28 (2.61)		11 (1.44)	38 (6.87)		13 (1.54)	36 (7.63)		5 (10.64)	40 (3.34)	
Duration of admission, (Mean ± SD)	13.55 ± 15.30	7.57 ± 9.50	< 0.001	6.20 ± 6.82	12.16 ± 14.43	< 0.001	6.18 ± 6.63	13.11 ± 15.19	< 0.001	17. 08 ± 16.57	8. 27 ± 10.57	< 0.001
Type of Ischemic stroke												
Large vessel	60 (21.98)	160 (12.44)	< 0.001	116 (12.50)	104 (16.48)	< 0.001	129 (12.75)	91 (16.64)	< 0.001	4 (6.15)	193 (13.80)	< 0.001
Other large	125 (45.79)	665 (51.71)		480 (51.72)	310 (49.13)		521 (51.48)	269 (49.18)		19 (29.23)	737 (52.68)	
Embolic	18 (6.59)	89 (6.92)		53 (5.71)	54 (8.56)		61 (6.03)	46 (8.41)		10 (15.38)	88 (6.29)	
Small	13 (4.76)	181 (14.07)		151 (16.27)	43 (6.81)		160 (15.81)	34 (6.22)		2 (0.08)	187 (13.37)	
Other	57 (20.88)	191 (14.85)		128 (13.790	120 (19.02)		141 (13.93)	107 (19.56)		30 (46.15)	194 (13.87)	

Prevalence of three-month mortality, excellent outcome, and functional independence was considerably associated with age ≥ 70, hypertension, diabetes, and AF. The type of stroke was related significantly to these favorable outcomes. Additionally, NIHSS, and duration of admission were associated with 3-month mortality, excellent outcome, and functional independence (Table 2).

Also, the prevalence of local hemorrhage was considerably more common among patients with AF, embolic stroke, and markedly lower in cases with small vessel stroke. Additionally, NIHSS and admission duration were associated with local hemorrhage (Table 2).

Table 3 presents the relationship between patient factors and various outcomes utilizing univariate and multivariate analyses. The factors related to the 3-month mortality, 3-month excellent outcome, 3-month functional independence, and local hemorrhage in univariate analysis were evaluated; further, the cases with these outcomes were compared with those without these outcomes (Table 3).

**Table 3:** Univariable and multivariable odds ratio (OR) of outcome of IVT ischemic stroke cases according to risk factors.

Variable	Mortality		Excellent functional outcome		Functional outcome 3 month		Local hemorrhage	
	Univariable model	Multivariable model	Univariable model	Multivariable model	Univariable model	Multivariable model	Univariable model	Multivariable model
	OR (95% CI)	OR (95% CI)	OR (95% CI)	OR (95% CI)	OR (95% CI)	OR (95% CI)	OR (95% CI)	OR (95% CI)
Vascular territory. PCS vs ACS	0.74 (0.37–1.46)	–	0.72 (0.44–1.19)	–	0.86 (0.52–1.42)	–	0.98 (0.30–3.21)	–
Age, median. ≥ 70 vs < 70	3.31 (2.50–4.39)	2.26 (1.59–3.23)	2.99 (2.42–3.68)	2.22 (1.69–2.91)	3.32 (2.67–4.13)	2.56 (1.92–3.41)	1.57 (0.96–2.59)	–
Gender. Female vs male	1.09 (0.84–1.42)	–	1.10 (0.90–1.35)	–	1.12 (0.91–1.38)	–	0.77 (0.47–1.28)	–
Smoking. Yes vs no	0.69 (0.43–1.12)	0.94 (0.52–1.71)	0.86 (0.62–1.21)	–	0.81 (0.57–1.16)	–	0.94 (0.40–2.23)	–
Atrial fibrillation. Yes vs no	1.79 (1.28–2.51)	1.99 (1.24–3.17)	1.68 (1.26–2.24)	1.15 (0.75–1.79)	1.70 (1.27–2.27)	1.37 (0.89–2.14)	3.27 (1.90–5.64)	2.75 (1.24–6.09)
Diabetes mellitus. Yes vs no	1.81 (1.37–2.39)	1.49 (1.06–2.08)	1.77 (1.43–2.18)	1.58 (1.21–2.07)	1.73 (1.39–2.15)	1.51 (1.14–2.01)	1.22 (0.73–2.05)	–
Hypertension. Yes vs no	1.96 (1.22–3.14)	1.56 (0.84–2.91)	1.48 (1.08–2.03)	1.18 (0.78–1.78)	1.73 (1.23–2.43)	1.43 (0.90–2.26)	0.70 (.036–1.35)	–
NIHSS								
6–14 vs < 6	4.19 (2.13–8.27)	3.30 (1.56–6.95)	2.92 (2.06–4.13)	2.03 (1.38–2.99)	2.94 (1.99–4.36)	1.92 (1.24–2.96)	1.89 (0.51–6.90)	1.65 (0.42–6.43)
15–24 vs < 6	19.23 (9.91–37.32)	10.80 (5.09–22.94)	12.62 (8.68–18.33)	6.16 (4.00–9.49)	14.48 (9.65–21.73)	6.50 (4.07–10.37)	9.07 (2.73–30.07)	5.22 (1.39–19.66)
≥ 25 vs < 6	23.18 (9.94–54.04)	14.27 (5.56–36.60)	18.59 (8.90–38.79)	8.35 (3.74–18.65)	21.77 (10.57–44.85)	10.04 (4.42–22.81)	12.58 (2.90–54.67)	7.25 (1.42–37.09)
Duration of admission (min)	1.04 (1.03–1.50)	1.02 (1.00–1.04)	1.07 (1.06–1.09)	1.06 (1.04–1.08)	1.08 (1.07–1.10)	1.07 (1.05–1.09)	1.06 (1.02–1.05)	1.02 (1.00–1.04)
Type of stroke								
Other large vessel vs large vessel	0.50 (0.35–0.71)	0.58 (0.37–0.90)	0.72 (0.53–0.97)	0.84 (0.56–1.26)	0.73 (0.54–0.99)	0.96 (0.63–1.46)	1.24 (0.42–3.70)	1.19 (0.38–3.74)
Embolic vs large vessel	0.54 (0.30–0.97)	0.22 (0.09–0.51)	1.14 (0.72–1.80)	0.80 (0.40–1.60)	1.07 (0.67–1.71)	0.60 (0.29–1.24)	5.48 (1.67–17.96)	2.49 (0.66–9.41)
Small vessel vs large vessel	0.19 (0.10–0.36)	0.47 (0.20–1.11)	0.32 (0.21–0.49)	0.66 (0.37–1.18)	0.30 (0.19–0.48)	0.70 (0.37–1.34)	0.52 (0.09–2.85)	1.28 (0.20–7.94)
Others vs large vessel	0.80 (0.52–1.21)	0.73 (0.41–1.32)	1.05 (0.73–1.50)	1.31 (0.77–2.22)	1.08 (0.74–1.55)	1.26 (0.73–2.17)	7.46 (2.58–21.58)	3.21 (0.99–10.42)

The odds of various outcomes were assessed for ten factors, including circulation, age, gender, cigarette smoking, AF, diabetes, hypertension, NIHSS score, admission duration, and stroke type. According to the results, all of these factors, except vascular territory, gender, and cigarette smoking, were significantly associated with 3-month mortality, excellent outcome, and functional independence in the univariate analysis.

### Three-month mortality outcome

Based on the multivariate logistic regression analysis with selection adjusted for covariates, the three-month mortality was significantly related to age ≥ 70 (OR 2.26, 95% CI 1.59–3.23), AF (OR 1.99, 95% CI 1.24–3.17), diabetes (OR 1.49, 95% CI 1.06–2.08), high NIHSS score (6–14, OR 3.30, 95% CI 1.56–6.95, NIHSS score 15–24, OR 10.80, 95% CI 5.09–22.94, NIHSS score > 24, OR 14.27, 95% CI 5.56–36.60 compared with subjects with NIHSS score ≤ 5), admission duration (OR 1.02, 95% CI 1.00–1.04), stroke type (other large vessels, OR 0.58, 95% CI 0.37–0.90 and embolic, OR 0.22, 95% CI 0.09–0.51 compared to subjects with the large vessel) (see Table 3).

### Three-month excellent outcome

According to the related results, the excellent outcome was significantly related to age ≥ 70 (OR 2.22, 95% CI 1.69–2.91), diabetes (OR 1.58, 95% CI 1.21–2.07), high NIHSS score (6–14, OR 2.03, 95% CI 1.38–2.99, NIHSS score 15–24, OR 6.16, 95% CI 4.00–9.49, NIHSS score > 24, OR 8.35, 95% CI 3.74–18.65 compared with subjects with NIHSS score ≤ 5) and admission duration (OR 1.06, 95% CI 1.04–1.08) (see Table 3).

### Three-month functional independence

The related results showed that functional independence (three-month) was significantly associated with age ≥ 70 (OR 2.56, 95% CI 1.92–3.411), diabetes (OR 1.51, 95% CI 1.14–2.01), high NIHSS score (6–14, OR 1.92, 95% CI 1.24–2.96, NIHSS score 15–24, OR 6.50, 95% CI 4.07–10.0.37, NIHSS score > 24, OR 10.04, 95% CI 4.42–22.81 compared with subjects with NIHSS score ≤ 5) and admission duration (OR 1.07, 95% CI 1.05–1.09) (see Table 3).

### Local hemorrhage

According to the results, local hemorrhage was significantly related to AF (OR 2.75, 95% CI 1.24–6.09), NIHSS score: 15–24, OR 5.22, 295% CI 1.39–19.66, NIHSS score > 24, OR 7.25, 95% CI 1.42–37.09 in comparison with subjects with NIHSS score ≤ 5) and admission duration (OR 1.02, 95% CI 1.00–1.04) (see Table 3).

## Discussion

This study investigated a population of Iranian ischemic stroke cases treated with IVT registered in SITS Registry. It followed their outcomes as three-month mortality, excellent outcome, functional independence, and local hemorrhage. It mainly aimed to compare outcomes in the two categories of PCS and ACS in three months and examine the other most important factors influencing three-month outcomes in IVT-treated stroke patients.

The results showed lower baseline NIHSS in PCS patients than ACS cases.

The NIHSS baseline score was significantly higher in ACS cases than PCS cases in the present work, similar to Macha, Kim, De Marchis, and Forster's study (on NIHSS baseline that was lower in PCS cases) and dissimilar to Tong's study^4,5,8,13,14^.

NIHSS has a weak point due to insensitivity to PCS signs, such as vertigo and imbalance, and could be untruly low in PCS cases compared to those of ACS. Thus, the severity of strokes in PCS and ACS cases is not accurate to the NIHSS score similarly. Accordingly, the findings about the severity of ACS and PCS may not be accurate.

In the current study, there was lower NIHSS and longer DNT in PCS cases. In the Cui et al. and Forster et al., onset to thrombolysis time (OTT) was longer in PCS patients^14,22^.

In the present work, age and OTT were not significantly different among ACS and PCS cases, similar to Tong et al. and De Marchis^4,13^. In the Kim study, ACS patients were older^8^.

Blood glucose level, WBC count, and AF history were not significantly different between the two circulations in our research as in Tong's study^3^.

In four studies, the female sex was higher in ACS cases, contrary to De Marchis and our study, which was the same in both sexes^4,5,8,9,13^.

In Cui et al., hypertension was observed more in PCS than ACS, which did not differ significantly between the two territories, unlike Tong et al., De Marchis, and the present work^4,13,22^.

Diabetes was less in PCS cases than ACS, as in Handelsmann et al. study and the opposite is true in Cui et al., Tong et al., and Kim et al. study. There was no difference in diabetes between ACS and PCS in the current study as in De Marchis^8,9,13,22^.

AF was higher in ACS cases in Cui, Handlesmann, Kim, and De Marchi's studies in contrary to Tongs' study, with higher prevalence in PCS cases. On the other hand, Macha et al. was as ours in that no difference was observed between the two circulation strokes^1,4,5,8,22,23^.

In Cui et al., there was no difference in poor prognosis between ACS and PCS cases as in our research, as well as Forster and WollenWeber et al.^14,15,22^.

In Tong et al., the outcome of 3 months was better in PCS cases, and mortality was not significantly different compared to ACS. Still, a lower rate of hemorrhage was seen in PCS cases^4^.

Hemorrhage in a base of vascular territory had a trend to ACS significantly in Tong, Das, and Forster et al. study and non-significantly in Macha et al. in contrast to our study^4,5,14,23^.

Kim et al. showed that minor PCS had worse functional independence after three months than minor ACS, especially in vertebrobasilar large vessel disease. On the other hand, we did not find a similar result in our cases (p-value = 0.618)^8^.

Sommer et al. showed a worse prognosis in PCS cases compared to ACS cases in unknown onset time or more than 4.5 h, as in KIM et al. in minor strokes, but in cases with arrival in less than 4.5 h, there was no difference between PCS and ACS cases as in our study^8,17^.

In a study on 18-month follow-up of IVT cases, there was 34.3% mortality, possibly making acceptable a mortality rate of 17.75% after a 3-month follow-up in the present work. PCS cases were about 7.00% of IVT therapy cases in this study, while in ours, it was about 4.60%; in other studies, more cases were reported compared to ours^4,5,9,20,22^. This difference could be due to the delay in diagnosis and admission of our PCS cases and fewer data collection.

Kim et al. found that worse outcomes in three months after minor PCS and ACS were related to baseline NIHSS (*P* < 0.001), but mortality in three months did not, confirming our results that the major predictor of disability is NIHSS and its increment directly affects 3-months disability^8^.

Lu et al. showed AF and NIHSS scores to be the primary indicators of poor prognosis in wake-up stroke cases treated with IVT. Local hemorrhage was directly associated with AF, and embolic stroke. On the other hand, it was conversely related to small vessel strokes, NIHSS score, and admission duration^24^.

Sun et al., in another study about minor strokes without IVT, found the primary NIHSS to be related to three months mRs^25^. Wang et al., Irvine et al., and Khazaei et al. showed NIHSS to be related directly to early neurologic deterioration^26–28^.

Finally, our research showed that the circulation was not an outcome predictor in ischemic strokes. The most important predictor was baseline NIHSS. The study showed no difference between ACS and PCS cases in the outcome of strokes, and IVT could be used in both circulations cautiously.

This study's strength was the number of stroke cases and the confidence of information gathered by neurologist supervision in all hospitals. On the other hand, it was the first study about the circulation effect on the outcome of Iranian stroke cases.

The limitations were incompleteness of information about the type of circulation and three months mRS of some cases (which were excluded from the study). Complications were not entirely registered in the registry. Also, some of the confounders as collateral and size of infarction were not collected in the registry.

Another limitation was number of PCS cases that was less than expected, possibly due to less awareness of stroke events in PCS cases both by family and emergency personnel. As registering the type of circulation is an optional item in SITS registry, there was incomplete data about circulation in many cases.

We offer a prospective study that focuses on circulation in IVT cases with or without poor outcome to examine how we could help them prevent the events after thrombolysis and achieve more detailed data about circulation in our cases.

## Conclusion

Overall, the present research shows that circulation is not an essential factor in determining the outcome of cases compared to NIHSS before and after thrombolysis. Higher NIHSS in both phases is related to poor outcomes and mortality. Other risk factors related to poor outcomes are diabetes, AF, admission duration, age, and stroke type.
